# The Accuracy of Individualized 3D-Printing Template-Assisted I^125^ Radioactive Seed Implantation for Recurrent/Metastatic Head and Neck Cancer

**DOI:** 10.3389/fonc.2021.664996

**Published:** 2021-03-31

**Authors:** Bin Qiu, Yuliang Jiang, Zhe Ji, Haitao Sun, Jinghong Fan, Weiyan Li, Yuxia Shao, Ping Jiang, Junjie Wang

**Affiliations:** ^1^Department of Radiation Oncology, Peking University Third Hospital, Beijing, China; ^2^Department of Radiation Oncology, National Cancer Center, National Clinical Research Center for Cancer, Cancer Hospital, Chinese Academy of Medical Sciences and Peking Union Medical College, Beijing, China; ^3^Engineering Research Center of Bone and Joint Precision Medicine, Ministry of Education, Beijing, China

**Keywords:** 3D-printing, radioactive seed implantation, head and neck cancer, brachytherapy, dosimetry

## Abstract

**Purpose:**

To evaluate the accuracy of individualized 3D-printing template-assisted I^125^ radioactive seed implantation (3D-PT assisted I^125^ RSI) for recurrent/metastatic head and neck cancer.

**Materials and Methods:**

From February 2017 to January 2020, clinical data of 41 patients (mean age, 58.5 ± 16.1 years; 28 males) with recurrent (48.8%)/metastatic (51.2%) head and neck cancer underwent individualized 3D-PT assisted I^125^ RSI under CT guidance in a single institute were retrospectively reviewed. Total 430 seed needles [mean, 10.5 (range 3–17) per patient] were inserted.

**Results:**

All seed needles were inserted manually in a single attempt with the technical success rate of 100% without major perioperative complications. The mean needle’s entrance deviation was 0.090 cm (95% Confidence Interval, 0.081–0.098). The mean intraoperative depth and angle of the needle were consistent with that of planned (6.23 ± 0.24 vs. 6.21 ± 0.24 cm, p = 0.903; 83.14 ± 3.64 vs. 83.09 ± 3.66 degrees, p = 0.985, respectively). The mean deviation between the needle’s planned and intraoperative depth and angle was 0.168 ± 0.024 cm and 1.56 ± 0.14 degrees, respectively. The postoperative dosimetry parameters, including D90, D100, V100, V150, V200, conformity index, external index, and homogeneity index, were all well-coordinated with planned dosimetry without significant difference (p = 0.515, 0.662, 0.958, 0.865, 0.872, 0.278, 0.456, and 0.989, respectively).

**Conclusions:**

Within the limitation of this study, individualized 3D-PT assisted I^125^ RSI may be accurate in obtaining favorable postoperative dosimetry for patients with recurrent/metastatic head and neck cancer.

**Clinical Trial Registration:**

[website], identifier [registration number].

## Introduction

Brachytherapy (BT) is a specific form of radiotherapy (RT) consisting of the precise placement of radioactive sources directly into or next to the tumor, which has the advantage of a rapid dose falling-off (1, 2). It is an optimal tool for delivering very high doses to the tumor focally while minimizing the probability of normal tissue complications (e.g., avoiding xerostomia), long-term functional and cosmetic outcomes usually are excellent (1, 2). Thus, both The Head and Neck Working Group of the European Brachytherapy Group and the American Brachytherapy Society recommended BT as one of the treatments for head and neck cancers (3, 4). As the mainstay of BT, I^125^ radioactive seed implantation (RSI) was reported to be safe and effective for recurrent/metastatic head and neck cancer as salvage therapy (5–8).

Owing to the dense critical organs and tissues (e.g., eyes, major vessels, and nerve) in the head and neck region, the accuracy of needle puncture and seed distribution during I^125^ RSI and postoperative dosimetry was extremely critical. The needle’s deviation (i.e., entrance point, angle, and depth deviation) between planned and intraoperative puncture may occur even under image guidance, which leads to mis-implantation of the I^125^ seeds and unnecessary radiation and damage to surrounding critical organs or tissues.

Using 3D-PT assistance for small nodules’ localization showed satisfied efficacy and safety and significantly simplified the localization procedure comparing with manual manipulation (9). Recently, an individualized 3D-printing template (3D-PT) was developed to facilitate I^125^ RSI to improve the accuracy and optimize postoperative dosimetry (5–8, 10–13). A 3D-PT-assisted technique significantly simplifies the procedure, improves the accuracy of implantation with higher dose in target volume margin, fewer needles and complications, and shortens the procedure duration (9, 14, 15). Furthermore, the postoperative dosimetry of 3D-PT assisted CT-guided I^125^ RSI may completely meet the requirements of preoperative plan as the seeds was precisely implanted (14, 16). While the accuracy of 3D-PT-assisted I^125^ RSI was not published for recurrent/metastatic head and neck cancer (11, 17). Here, the study aims to evaluate the accuracy of needle puncture and postoperative dosimetry of individualized 3D-PT-assisted I^125^ RSI for patients with recurrent/metastatic head and neck cancer in a single institute.

## Materials and Methods

### Study Design

The retrospective study was approved by our institutional review board (IRB) and the requirement to obtain written informed consent was waived. The electronic database of a single institute was searched and reviewed to identify eligible patients. Forty-one patients who underwent 3D-PT-assisted I^125^ RSI under CT guidance for the treatment of recurrent/metastatic head and neck cancer between February 2017 and January 2020 were included. The indications for I^125^ RSI were recurrent/metastatic head and neck cancer after surgery/external beam radiotherapy (EBRT)/first-line system therapy in patients who are not eligible for salvage surgery/EBRT (metastatic head and neck cancer refer to a secondary cancer that occurred in the head and neck region regardless of the primary site). The contraindications were as follows: (i) Active infection; (ii) The diameter of largest tumor > 7 cm or any active concomitant distant cancer; (iii) Karnofsky Performance Score < 70 or predicted life span < 3 months; (iv) Approach of I^125^ RSI deemed not available on preoperative CT/MRI; (v) International normalized ratio > 2, and (vi) Pregnancy/mental disorder or any somatic comorbidities of clinical concern.

**Figure 1:**
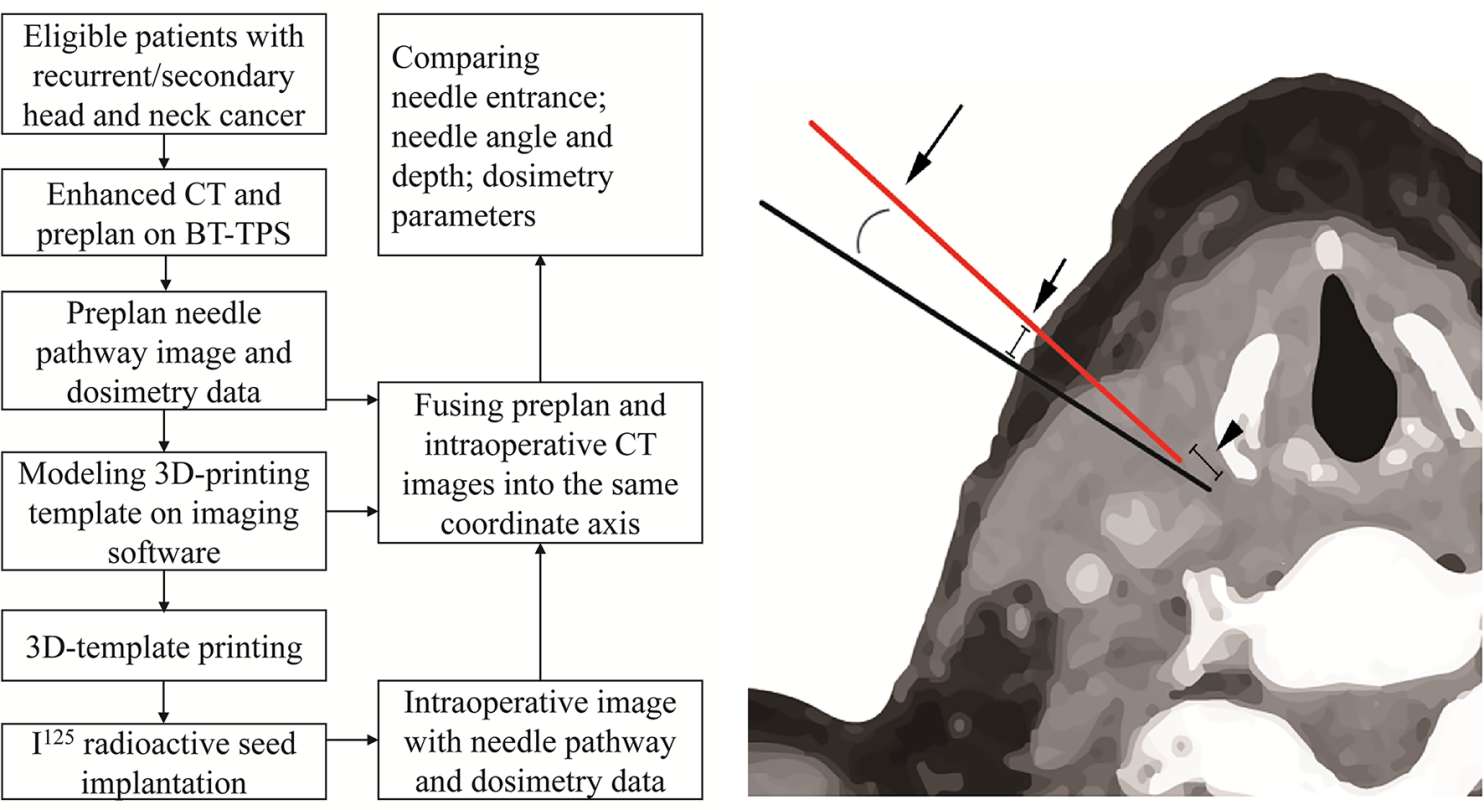
The flow chart of the study and measurement of the deviation between planned needle (black) and intraoperative needle (red) after fusing the planned and intraoperative CT images into the same coordinate axis on brachytherapy treatment planning system (BT-TPS): entrance point deviation (short arrow), depth deviation (arrowhead), and angle deviation (long arrow).

The planned and intraoperative needle’s entrance deviation, angle, and depth were extracted from the BT Treatment Planning System (BT-TPS) after fusing the planned and intraoperative CT images on the same coordinate axis. The mean needle’s entrance deviation was calculated as the superficial distance between the planned needle’s entrance point and the actual intraoperative needle’s entrance point on CT images. The needle’s depth was calculated as the depth from the tip of the needle to the template surface when the needle is deemed in place before the seed implantation. The needle’s angle was calculated as the angle between the needle and the horizontal axis. The flow chart of the study and measurement of the deviation between planned and intraoperative needle puncture (i.e., entrance point, angle, and depth deviation) is shown in **Figure 1**. The technical success rate, planned and postoperative number of needles and seeds, and dosimetry parameters, including the prescription dose, gross tumor volume (GTV), D90, D100, V100, V150, V200, conformity index (CI), external index (EI), and homogeneity index (HI) were also recorded and compared. Subgroups analysis by cancer type (recurrent/metastatic) and implantation site (head/neck, bounded by the connecting line of the lower margin of the jaw, the mandibular angle, the tip of the mastoid process, superior nuchal line, and the external occipital carina) were conducted.

### Definitions

The planned data refers to data of preoperative planning or only intraoperative re-plan data (if available). Technical success is defined as successful needle insertion and implantation of I^125^ seed in the targeted volume per that of planned. D90 and D100 refer to the dose delivered to the 90% or 100% of GTV, respectively. V100, V150, and V200 refer to the percentage of GTV receiving 100% or 150% or 200% of the prescription dose, respectively. CI, EI, and HI were defined according to the International Commission on Radiation Units and Measurements (ICRU) Report 62 (18). Complications were defined as a minor (grade 1-2) and major (≥ grade 3) according to Common Terminology Criteria for Adverse Events v 4.0. Local-progression free survival was defined as the duration from RSI until local implantation site disease progression or death from any cause. Overall survival was defined as the interval between RSI and death from any cause.

### Study Population

Total 41 patients (mean age, 58.5 ± 16.1; range, 10–87 years) with recurrent/metastatic head and neck cancer were included. Majority of the patients were males (n = 28, 68.3%). Over half of the cancers were located in the neck region (n=22, 53.7%). Recurrent head and neck cancer (n=20, 48.8%) included oral carcinoma (n=6, 14.6%), oropharyngeal cancer (n=2, 4.9%), laryngeal cancer (n=2, 4.9%), thyroid cancer (n=2, 4.9%), orbital rhabdomyosarcoma (n=2, 4.9%), and other cancers (n= 6, 14.6%). Metastatic head and neck cancer (n=21, 51.2%) included lymphatic metastasis (n=19, 46.3%) [derived from lung cancer (n = 4, 9.8%), esophageal cancer (n=3, 7.3%), nasopharynx cancer (n=3, 7.3%), oral carcinoma (n=3, 7.3%), laryngo-carcinoma (n=1, 2.4%), thymic carcinoma (n=1, 2.4%), breast cancer (n=1, 2.4%), cervical cancer (n=1, 2.4%), and lymphatic metastasis of unknown (n=2, 4.9%)] and brain metastasis (n=2, 4.9%) derived from lung cancer. All the patients received previous treatments. The mean deep of the tumor was 8.1 ± 2.8 cm, which was calculated as the maximum vertical distance from the deepest point of the tumor to the skin. The mean gross volume before RSI was 20.5 ± 16.6 cm^3^, as described in **Table 1**.

**Table 1:** Clinical characteristics of the patients.

Item	n (%)
Age (Years)	58.5 ± 16.1
Sex	
Male	28 (68.3)
Female	13 (31.7)
Recurrent cancer	20 (48.8)
Oral carcinoma	6 (14.6)
Oropharyngeal cancer	2 (4.9)
Laryngo-carcinoma	2 (4.9)
Thyroid cancer	2 (4.9)
Orbital rhabdomyosarcoma	2 (4.9)
Others	6 (14.6)
Secondary cancer	21 (51.2)
Lymphatic metastasis	19 (46.3)
Cerebral metastasis	2 (4.9)
Previous treatment	
Chemoradiotherapy	14 (34.1)
Surgery	7 (17.1)
Radiotherapy	6 (14.6)
Chemotherapy	5 (12.2)
Surgery + Chemoradiotherapy	5 (12.2)
Surgery + Radiotherapy	4 (9.8)
Implanted site	
Head region	19 (46.3)
Neck region	22 (53.7)
Deep of the tumor (cm)	8.1 ± 2.8
Gross volume of the tumor (cm^3^)	20.5 ± 16.6

Plus-minus data = mean ± standard deviation; number in parentheses = percentage of patients.

### Preoperative Planning

All patients underwent contrast-enhanced CT with 2.5-mm or 5-mm (rarely, for large tumors only) resolution within 2–3 days before RSI. All patients were fixed with a bow cap/vacuum pad at suitable point according to the lesion location and facilitation for RSI and then marked with surface positioning line. Then the CT images were transferred into the BT-TPS (Beijing Feitian Industries Inc and Beijing University of Aeronautics and Astronautics, Beijing, China). The preoperative planning was then established by defining GTV and adjacent organs at risk (OARs) and determining prescription dose according to an expert consensus on I^125^ RSI (19), commonly between 110-160Gy. The radioactivity of the I^125^ seeds is usually 0.4–0.7 mCi. The distribution of the seeds and the needles’ pathway were determined after verifying the dose calculations of the GTV and OARs.

**Figure 2:**
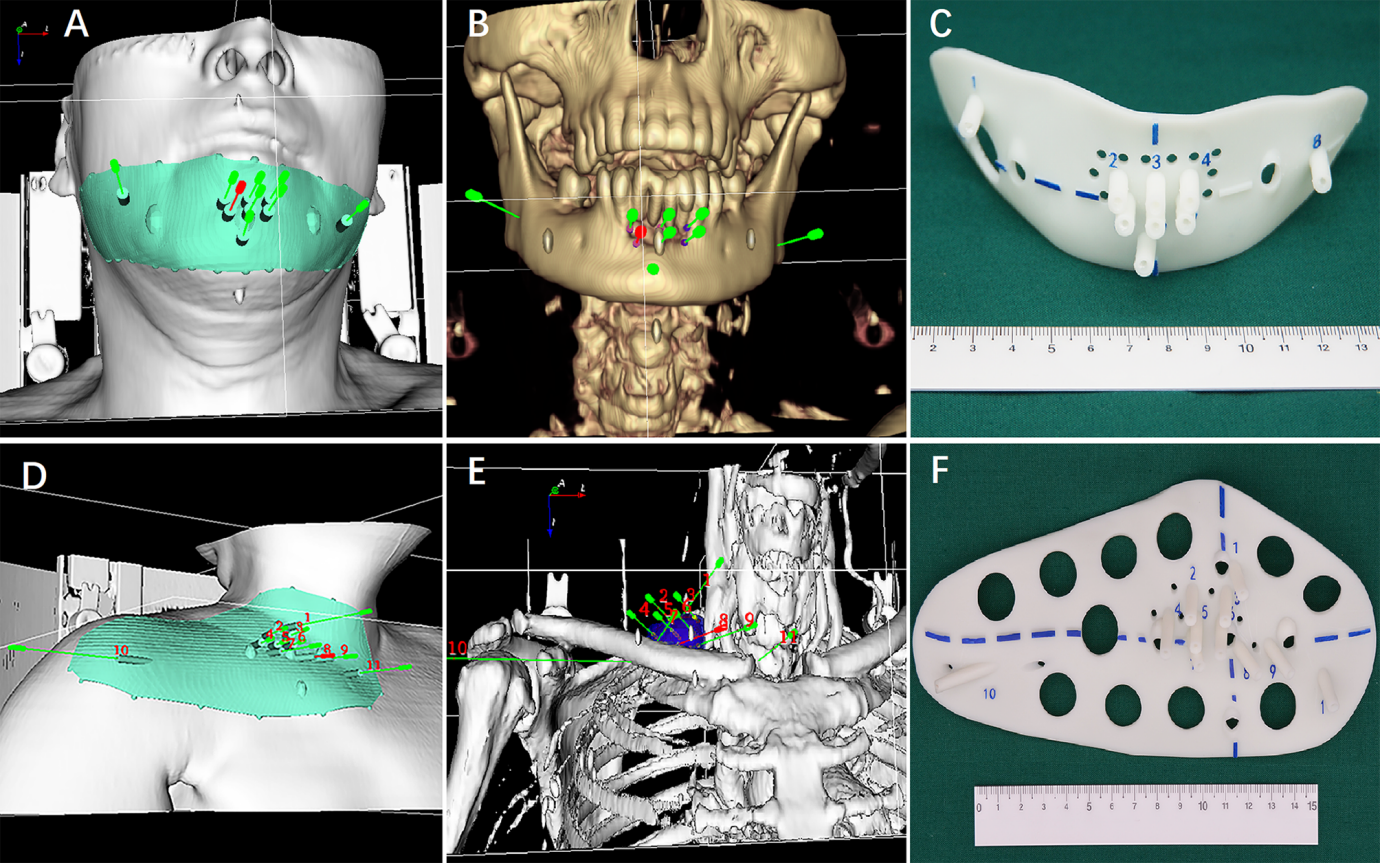
**(A, B and D, E)** Digital modeling of the individualized 3D-printing template (3D-PT) for head and neck; **(C, F)** The 3D-PT (3 mm thickness) with entrance aisle for an 18-gauge needle (the entrance aisle and the needle was perfectly matched; therefore, the needle’s angle was ensured).

The individualized preoperative planning data in the BT-TPS was then transferred into 3D imaging and reverse engineering software for digital modeling of individualized 3D-PT. Subsequently, the modeling data was optimized using Magics 19.01 software (Materialise Company, Belgium), and the individualized 3D-PT was finally produced using 3D light-cured rapid-forming printer RS6000 (Shanghai Liantai 3D Technology Company, Shanghai, China). The 3D-PT with 3 mm thickness contained individualized information such as body-surface characteristics of the target region, localization markers, and entrance aisle for 18-gauge needle (14) (the entrance aisle and the needle were perfectly matched, therefore, the needle’s angle was ensured) (**Figure 2**).

### I^125^ RSI Procedure

**Figure 3:**
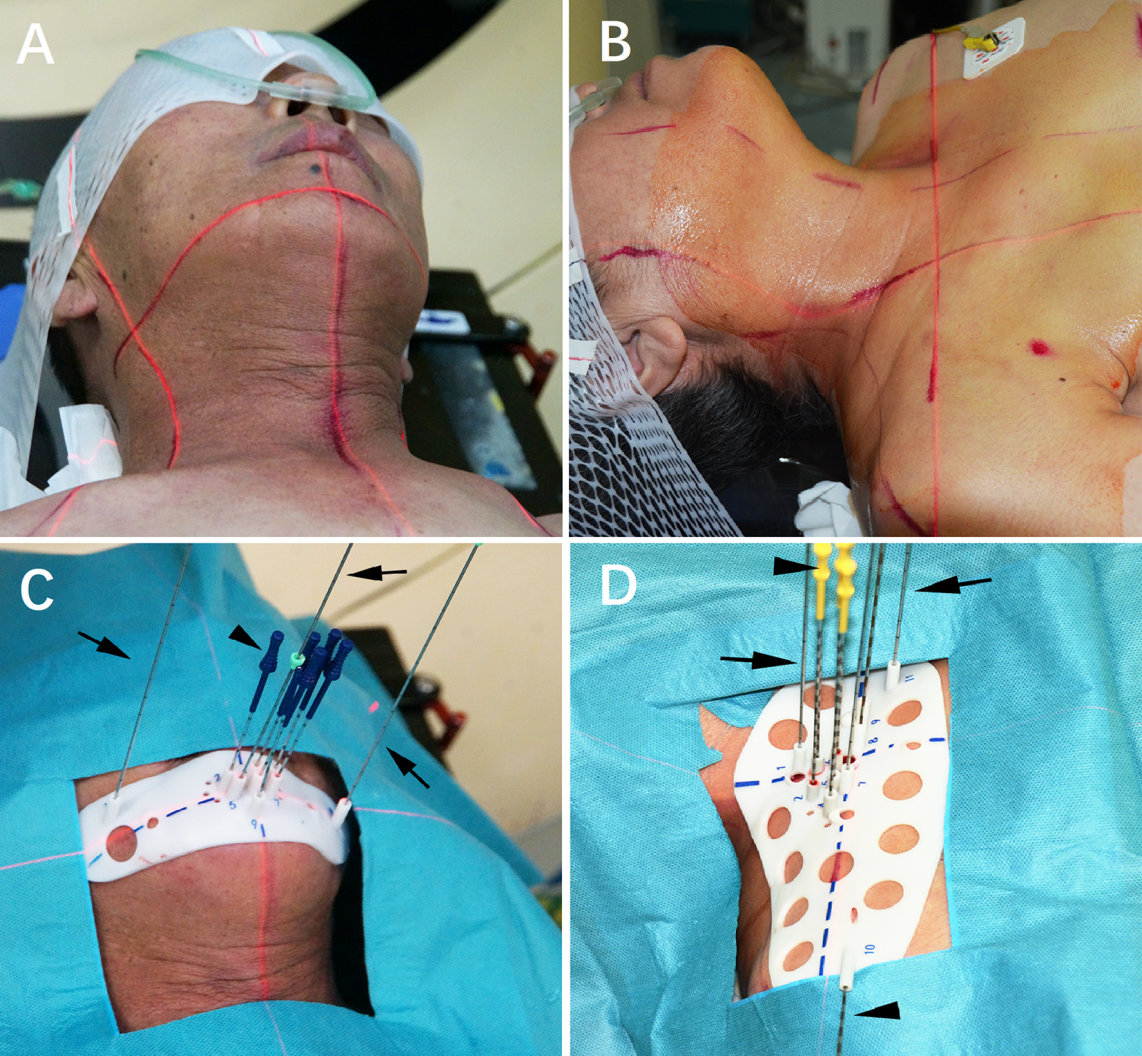
I^125^ radioactive seed implantation. **(A, B)** Patients were fixed; **(C, D)** Individualized 3D-printing template (3D-PT) was aligned.

**Figure 4:**
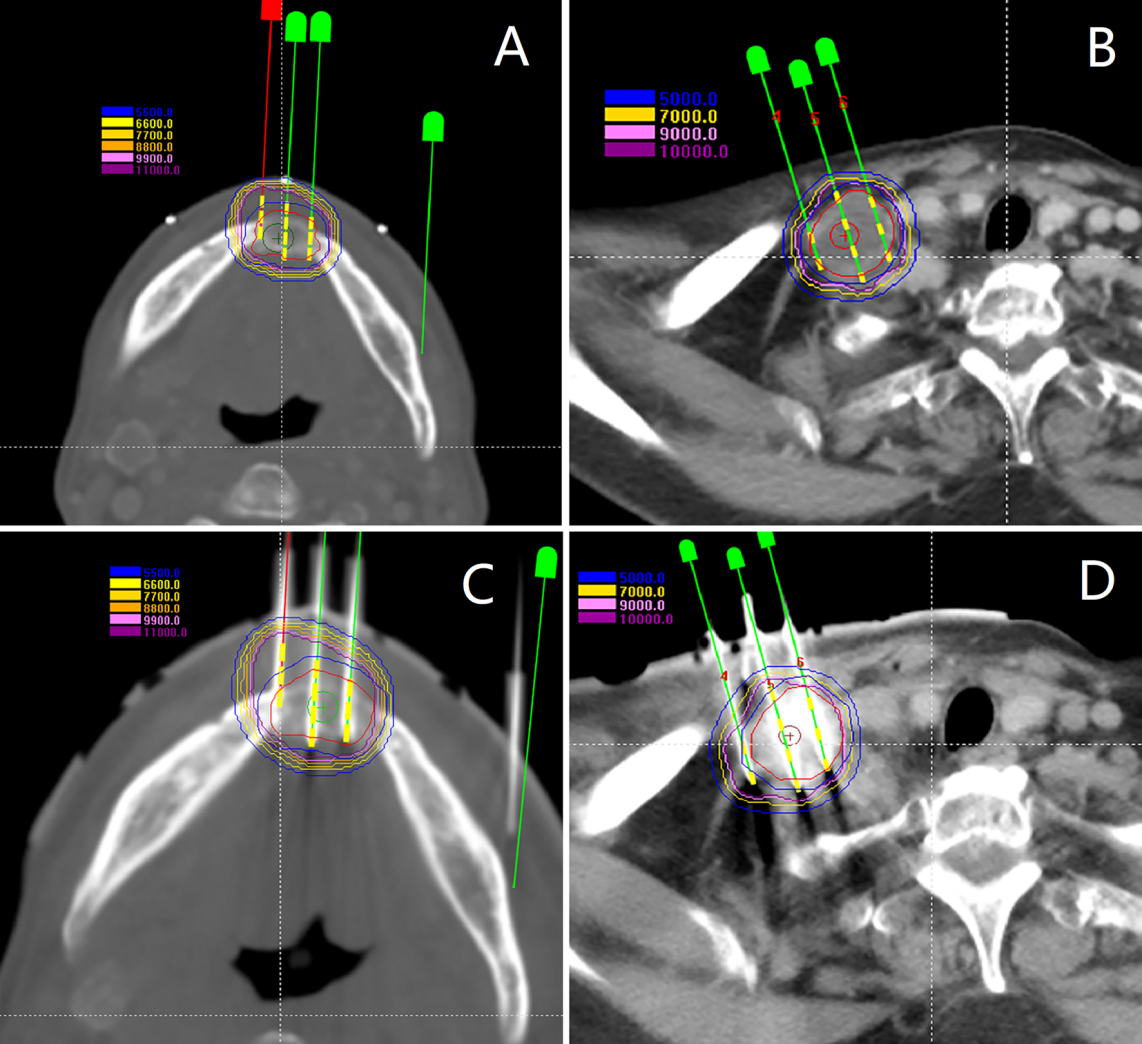
**(A, B)** Intraoperative plan and the additional needle was added; **(C, D)** I^125^ seeds were implanted.

Three experienced doctors (all>5 years’ experience) performed all I125 RSI procedures under local anesthesia with CT guidance. After skin preparation and sterilization, the 3D-PT was aligned to the target region according to the body-surface characteristics, reference line on the 3D-PT, surface positioning line, and positioning laser (**Figure 3A, B**). Then CT scan was performed. Identified malposition of the 3D-PT between the preoperative planning and the current CT image was adjusted and then 2–3 locking needles (18-gauge) followed by the seed implantation needles (18-gauge) were percutaneously inserted *via* the preoperative planning aisle on the 3D-PT (i.e., each needle’s depth), (**Figure 3C, D**). After all the needles were deemed in place, the I^125^ seeds were implanted and delivered using the Mick applicator. Seeds were implanted during the needle retreating with a 0.5/1.0 cm interval according to the preoperative planning or intraoperative re-plan (**Figure 4**). All RSI procedures were performed following relevant guidelines and regulations, as also described in the previously published study (5, 6).

### Postoperative Verification

**Figure 5:**
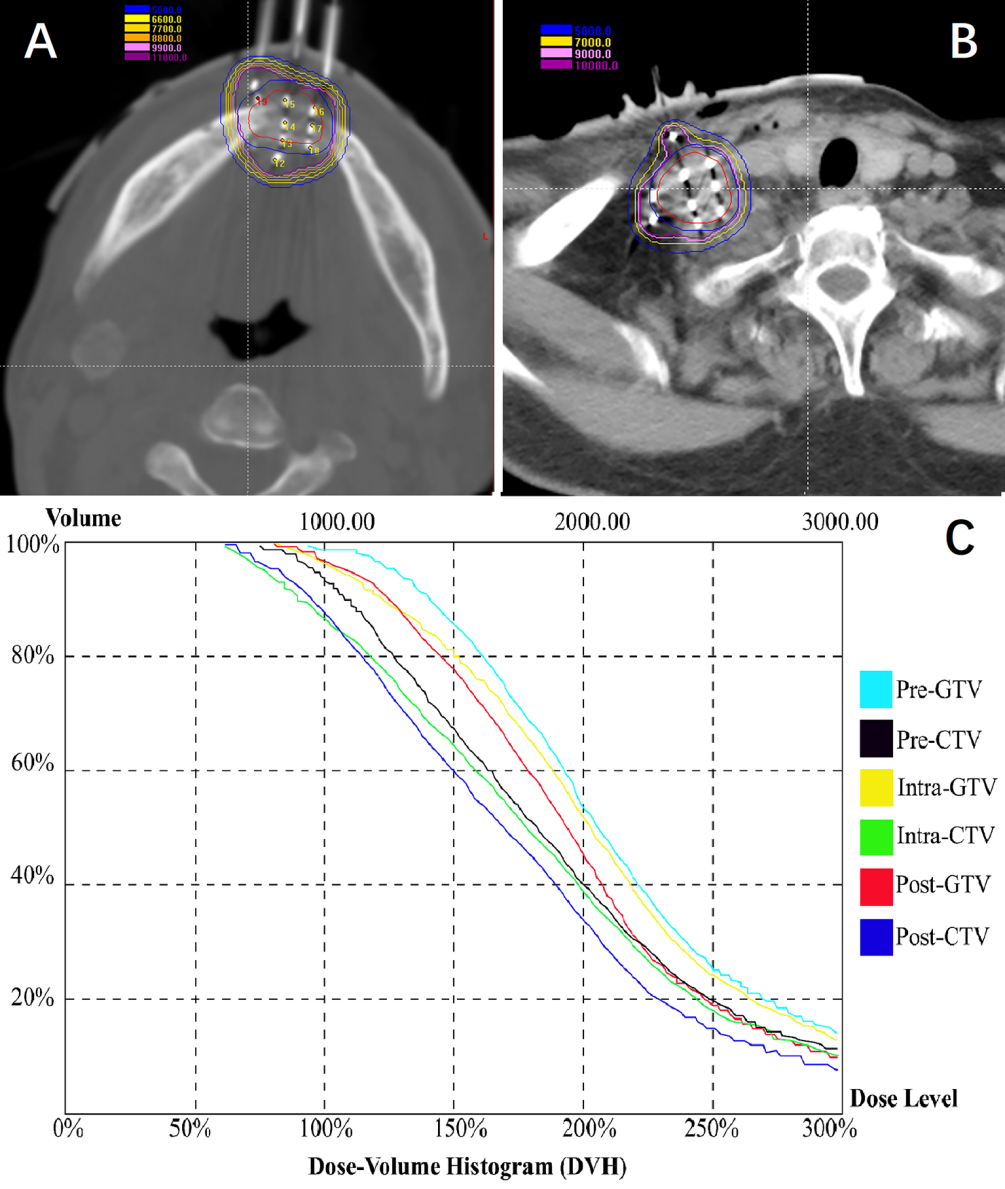
Patients were re-evaluated immediately with a CT scan after I^125^ radioactive seed implantation. **(A, B)** Validation of postoperative distribution of the I^125^ seeds; **(C)** The dose-volume histogram of preoperative planning, intraoperative re-plan, and postoperative validation.

Total 428 [mean, 10.4 (range 3–18) per patient] seed needles were planned while 430 [mean, 10.5 (range 3–17)] seed needles were inserted during RSI. Eight patients (19.5%) underwent intraoperative re-plan and adjusted the number of inserted needles, these patients’ intraoperative re-plan dosimetry data were analyzed together with the remaining patients’ preoperative planning dosimetry data as planned dosimetry. All patients were re-evaluated immediately with a CT scan after I^125^ RSI to validate the postoperative distribution of the I^125^ seeds and rule out potential perioperative complications. Then, the CT images were transferred to BT-TPS to verify postoperative dosimetry (**Figure 5**). Dosimetry parameters including D90, D100, V100, V150, V200, CI, EI, and HI were evaluated.

### Follow-Up

Clinical outcomes were routinely followed, the evaluation of tumor response was conducted based on the CT/MRI images obtained 2-3 month after RSI according to the Response Evaluation Criteria in Solid Tumors (RECIST) v1.1 (20). Then follow-up at a 3–6-month interval was executed after RSI.

### Statistical Analysis

Continuous variables were compared using a paired t-test between pre-planning data and intraoperative data/post verification data. As 84.2% of cancer in the head were recurrent cancer and 81.8% of cancer in the neck were metastatic cancer, subgroups analysis only by cancer type and implantation site were further conducted in multivariate analysis using a linear regression model. A 2-sided p-value < 0.05 was considered as statistically significant difference. Statistical analyses were performed using SPSS software (version 26.0; SPSS, Chicago, IL, USA).

## Results

### Procedure Details

All seed needles were inserted manually in a single attempt, the technical success rate was 100%. The mean planned and implanted seeds per patient were 42.6 (range, 11–85) and 44.4 (range, 12–85), respectively. The prescription dose was 90–170 (mean, 136.1 ± 7.7) Gy and intraoperative GTV was 1.2–85.2 (mean, 20.5 ± 5.1) cm³. Pain (26.8%) and a small amount of bleeding (78%) at the puncture site was seen in some of the patients and all were self-healing after RSI. No major perioperative complications (e.g., mis-implantation of radiative seeds, adjacent main arteriovenous, or other critical organ damage) were observed.

### Accuracy of Needle Puncture and Postoperative Dosimetry

Of the 430 inserted needles, the mean needle’s entrance deviation was 0.090 cm (95% Confidence Interval, 0.081–0.098; range, 0–0.350 cm). The mean needle’s intraoperative depth and angle were consistent with that of planned (6.23 ± 0.24 vs. 6.21 ± 0.24 cm, p = 0.903; 83.14 ± 3.64 vs. 83.09 ± 3.66 degrees, p=0.985, respectively). The mean needle’s depth and angle deviation between planned and intraoperative data were 0.168 ± 0.024 (range, 0–0.400) cm and 1.56 ± 0.14 (range, 0–7.20) degrees, respectively. The planned D90 and D100 were well coordinate with that of postoperative (160.0 ± 6.2 and 156.3 ± 9.1Gy, p = 0.515; 83.6 ± 7.1 and 80.8 ± 10.0Gy, p = 0.662, respectively). Along with other dosimetry data, the planned and postoperative V100, V150, and V200 were 19.4 ± 4.8 and 19.2 ± 4.9 (p = 0.958), 15.1 ± 3.8 and 14.6 ± 3.7 (p = 0.865), and 9.9 ± 2.8 and 9.5 ± 2.8 (p = 0.872), respectively, and no significant difference was observed. The planned and postoperative CI, EI, and HI were 0.52 ± 0.04 and 0.49 ± 0.04 (p = 0.278), 0.91 ± 0.20 and 1.04 ± 0.25 (p = 0.456), and 0.31 ± 0.14 and 0.31 ± 0.15 (p = 0.989), respectively, with no significant difference (**Table 2**).

**Table 2:** Analysis of pre-planning and intraoperative/post-plan parameters.

Parameter	Planned	Intraoperative	Post-plan	p value
Depth of needle	6.21 ± 0.24	6.23 ± 0.24		0.903
Angular of needle	83.09 ± 3.66	83.14 ± 3.64		0.985
D90	160.0 ± 6.2		156.3 ± 9.1	0.515
D100	83.6 ± 7.1		80.8 ± 10.0	0.662
V100	19.4 ± 4.8		19.2 ± 4.9	0.958
V150	15.1 ± 3.8		14.6 ± 3.7	0.865
V200	9.9 ± 2.8		9.5 ± 2.8	0.872
CI	0.52 ± 0.04		0.49 ± 0.04	0.278
EI	0.91 ± 0.20		1.04 ± 0.25	0.456
HI	0.31 ± 0.14		0.31 ± 0.15	0.989

Plus-minus data = mean ± 1.96 standard error; D90 and D100 refer to the dose delivered to the 90% or 100% of gross tumor volume and V100, V150, and V 200 refer to the percentage of gross tumor volume receiving 100% or 150% or 200% of prescription dose, respectively; CI, Conformity index; EI, External index; HI, Homogeneity index.

### Subgroup Analysis

In the univariate analysis, the needle’s entrance deviation in patients with recurrent cancer was significantly larger than patients with metastatic cancer (0.107 ± 0.012 vs. 0.072 ± 0.012 cm, p < 0.001) and was comparable in patients with implantation in the region of the head and that of the neck (0.089 ± 0.011 vs. 0.090 ± 0.013 cm, p = 0.938). The mean deviation of needle’ depth had no significant difference between patients with recurrent and metastatic cancers (0.169 ± 0.041 vs. 0.167 ± 0.026 cm, p = 0.951) or between patients with implantation in the region of the head and that of the neck (0.152 ± 0.043 vs. 0.182 ± 0.025 cm, p = 0.224). In contrast, the mean deviation of the needle’ angle was smaller in patients with recurrent cancers than with metastatic cancers (1.18 ± 0.19 vs. 1.94 ± 0.19 degrees, p < 0.001) and smaller in patients with implantation in the region of the head than that of the neck (1.25 ± 0.19 vs. 1.84 ± 0.19 degrees, p < 0.001) (**Table 3**).

**Table 3:** Subgroup analysis of pre-plan and intraoperative parameter deviation (univariate analysis).

Parameter	Cancer type		p value	Implantation site		p value
	Recurrent	Secondary		Head	Neck	
Entrance deviation	0.107 ± 0.012 cm	0.072 ± 0.012 cm	<0.001	0.089 ± 0.011 cm	0.090 ± 0.013 cm	0.938
Depth deviation	0.169 ± 0.041 cm	0.167 ± 0.026 cm	0.951	0.152 ± 0.043 cm	0.182 ± 0.025 cm	0.224
Angular deviation	1.18 ± 0.19 degrees	1.94 ± 0.19 degrees	<0.001	1.25 ± 0.19 degrees	1.84 ± 0.19 degrees	<0.001

In the multivariate analysis using a linear regression model including both cancer type and implantation site, the variance test of linear regression for needle’s entrance deviation had statistical significance (p < 0.001). The needle’s entrance deviation was significantly different between patients with recurrent cancers and patients with metastatic cancers (p < 0.001) and was significantly different between patients with implantation in the region of the head and that of the neck (p < 0.001). However, variance test of the linear regression for deviation of needle’s depth and angle both had no statistical significance (p = 0.065 and p = 0.092, respectively).

### Long-Term Safety

Until January 2021, 2 major and 3 minor complications occurred during a median follow-up duration of 19 months. The 2 major complications were mucosal ulcer and skin ulcer. The mucosal ulcer was observed in a patient with nasopharynx cancer who previously received EBRT and died 2 months after RSI owing to massive hemorrhage of the ulcer. The skin ulcer was observed in the left mandibular of a previously irradiated patient with oral carcinoma and finally formed fistula, and the patients died 5 months after RSI owing to tumor progression. The 3 minor complications were radiodermatitis (n=2) and skin pigmentation (n=1) and all improved without additional treatment.

### Clinical Efficacy

The clinical efficacy of patients with recurrent head and neck cancer was analyzed, 19 (94.7%) patients were available and 1 (5.3%) patient lost to follow-up. Among the 19 patients, 1 (5.3%) complete remission (CR), 11 (57.9%) partial remission (PR), 5 (26.3%) stable disease (SD), and 2 (10.5%) progressive disease (PD) were observed. The local control rate (CR+PR) was 63.2%. Until January 2021, 6 patients were still alive. The estimated median local-progression free survival was 7 months [interquartile range (IQR), 6– - months] and the estimated median overall survival was 12 months (IQR, 6–24 months).

## Discussion

The present study indicated that the accuracy of needle puncture and postoperative dosimetry was satisfied for individualized 3D-PT-assisted I^125^ RSI in patients with recurrent/metastatic head and neck cancers. Since the introduction of 3D-PT in clinical practice, few studies investigated the accuracy of needle puncture during 3D-PT-assisted needle-related interventions (9, 17). As revealed by a non-inferiority randomized clinical trial that enrolled 200 patients for localizing small pulmonary nodules (9), localizer deviation did not significantly differ between the 3D-PT-assisted group and CT-guided group (mean, 8.7 vs. 9.6 mm; p = 0.36). The mean procedural durations were 7.4 minutes for the 3D-PT-assisted group and 9.5 minutes for the CT-guided group (P < 0.001). The mean CT-related radiation dose was 229 mGy × cm in the 3D-PT-assisted group and 313 mGy × cm in the CT-guided group (p < .001) (9), indicating that the use of the 3D-PT for placement of pulmonary localizer showed efficacy and safety that were not substantially worse than those with the CT-guided alone, while significantly simplifying the procedure and decreasing patient CT-related radiation exposure.

For patients with head and neck cancers, the relatively stable craniocerebral structure may fascinate the usage of individualized 3D-PT, and the deviation of needle puncture during RSI may be prone to be smaller than that of localizing pulmonary nodules. Ming-Wei Huang et al. (17) reported 25 patients with head and neck tumors implanted with I^125^ radioactive seeds under the assistance of 3D-PT. The mean entrance deviation for all inserted needles was 1.18 ± 0.81 mm varying from 0.857 ± 0.545 to 1.930 ± 0.843 mm at different sites and was significantly smaller in the parotid and maxillary regions (belong to head region) that are significantly smaller than those of localizing pulmonary nodules mentioned above and similar to those reported here (0.81–0.98 mm). In the present study, the needle’s entrance deviation was also significantly different in patients with implantation in the head and neck region and patients with recurrent cancer and metastatic cancer in the multivariate analysis but was only larger in patients with recurrent cancer in univariate analysis. Meanwhile, in the study by Ming-Wei Huang et al. (17), the mean angle deviation was 2.08 ± 1.07 degrees varying from 1.85 ± 0.93 to 2.73 ± 1.18 degrees at different sites and was significantly larger (indicating less accurate placement) in the sub-mandibular and upper neck area (neck region), than in the other regions (head region), which also seems similar to that reported here (1.56 ± 0.14 degrees). In the current study, the needle’s angle deviation was larger in patients with metastatic cancer than recurrent cancers in univariate analysis. However, in multivariate analysis, both planned and intraoperative deviation of needles’ angle and depth had no statistical significance in both cancer types. Therefore, further high-quality study is needed before drawing the conclusion on the accuracy of 3D-PT-assisted RSI by cancer type or implantation site.

As for dosimetry profile, in the above study of Ming-Wei Huang et al. (17), the D90 was larger than that of planned and ranged from 122Gy to 198Gy (mean 163.8 ± 22.6Gy), which seems higher than that reported here (range, 90–170; mean, 136.1 ± 7.7 Gy). The V100 was larger than 95% and the V150 was less than 50% in all patients and other planned and postoperative dosimetry data (e.g., V150, V200, CI, EI, and HI) were not reported in their study. In a study by Ji Z et al. (14), comparison between the dose distributions of postoperative data with planned data for 3D-PT-assisted RSI yielded enrollment of 14 patients with malignant tumors (majority located in the pelvic cavity). The average postoperative D90, V100, and V150 were smaller than the planned ones, and average postoperative V200 and the minimum peripheral dose of GTV were larger than the planned ones. However, there was no statistical difference in any of these parameters between the two groups except for V100 (p=0.027). Sun et al. (16) compared the dosimetry data between preoperative planning and postoperative verification in 3D-PT-assisted CT-guided RSI for thoracic tumors. All of the included dosimetry parameters coordinated slightly, while the difference was also not statistically significant (all p > 0.05). Liang et al. (13) reported the dosimetry accuracy of 3D-PT-assisted I^125^ RSI for the treatment of cervical lymph node metastasis in 15 patients. There was also no significant difference for all the parameters (D90, V90, V100, and V150) between preoperative planning and postoperative verification (all p > 0.05). Similarly, as also revealed in the current study, the postoperative dosimetry has completely met the planned requirements for 3D-PT-assisted RSI without significant difference.

Ji et al. (5) reported 101 patients with recurrent head and neck cancer after EBRT who received CT-guided I^125^ RSI. The local control rate was 60.7%, which is similar to that reported here (63.2%). The median survival was 15 months, which seems inferior to that reported here (24 months). Furthermore, major and minor skin or mucosal complications occurred in 10 patients (9.9%) and 16 patients (15.8%), respectively, during a median follow-up of 12.2 months, which may be inferior to that reported here. In our study, 2 major and 3 minor complications occurred during a median follow-up of 19 months. The major complication was all observed in previously irradiated patients. Skin or mucosal toxicity was also reported in patients with ultrasound-guided I^125^ RSI for head and neck cancers with a rate of 2.5% for major and 17% for minor complications (21). Therefore, I^125^ RSI may be used with caution in patients with superficial tumors who had previously received EBRT.

The present study has several limitations. First, this was a retrospective study and therefore prone to potential selection bias. Second, the absence of a control group limits evaluation of the superiority of 3D-PT-assisted CT-guided RSI over barehanded CT- guided RSI. Third, the needle’s depth and angle were calculated after fusing the planned and intraoperative CT images into the same coordinate axis on BT-TPS, which suffered from potential fusion error. However, this is the only way to compare planned data with intraoperative data. Finally, in the subgroup analysis for implantation site, further refined sub-region classification, e.g., the parotid and masseter region, maxillary and paranasal region, the retromandibular region, and submandibular and upper neck region, was not applied in the present study, limited by the power of statistics in such small group of patients. In conclusion, within the limitation of this study, individualized 3D-PT-assisted I^125^ RSI may be accurate in obtaining favorable postoperative dosimetry for patients with recurrent/metastatic head and neck cancer.

## Data Availability Statement

The raw data supporting the conclusions of this article will be made available by the authors, without undue reservation.

## Ethics Statement

The studies involving human participants were reviewed and approved by Peking University Third Hospital Medical Science Research Ethics Committee. The ethics committee waived the requirement of written informed consent for participation.

## Author Contributions

BQ, PJ, and JJW conceived and designed the study. BQ, YL J, ZJ, HTS, JHF, WYL, and YXS performed the data collection and are responsible for statistical analysis. BQ and PJ wrote the paper. JJW reviewed and edited the manuscript. All authors contributed to the article and approved the submitted version.

## Funding

National Key Research and Development Plan of China (Grant No. 2019YFB1311300) to JW supports the implementation (e.g., labor cost and data collection) and publication of the project.

## Conflict of Interest

The authors declare that the research was conducted in the absence of any commercial or financial relationships that could be construed as a potential conflict of interest.

## Abbreviations

BT, brachytherapy; CT, Computerized tomography; CI, conformity index; EBRT, external beam radiotherapy; EI, external index; GTV, gross tumor volume; HI, homogeneity index; RT, radiotherapy; RSI, radioactive seed implantation; MRI, Magnetic resonance imaging; OARs, organs at risk; TPS, Treatment planning system; 3D-PT, 3D-printing templates.
